# Good microbes, bad genes? The dissemination of antimicrobial resistance in the human microbiome

**DOI:** 10.1080/19490976.2022.2055944

**Published:** 2022-03-25

**Authors:** Alexander Crits-Christoph, Haley Anne Hallowell, Kalia Koutouvalis, Jotham Suez

**Affiliations:** W. Harry Feinstone Department of Molecular Microbiology and Immunology, Johns Hopkins Bloomberg School of Public Health, Baltimore, Maryland, USA

**Keywords:** Microbiome, resistome, antibiotics, probiotics, fecal microbiome transplantation (FMT), metagenomics, antibiotic resistance genes (ARG), antimicrobial resistance (AMR)

## Abstract

A global rise in antimicrobial resistance among pathogenic bacteria has proved to be a major public health threat, with the rate of multidrug-resistant bacterial infections increasing over time. The gut microbiome has been studied as a reservoir of antibiotic resistance genes (ARGs) that can be transferred to bacterial pathogens via horizontal gene transfer (HGT) of conjugative plasmids and mobile genetic elements (the gut resistome). Advances in metagenomic sequencing have facilitated the identification of resistome modulators, including live microbial therapeutics such as probiotics and fecal microbiome transplantation that can either expand or reduce the abundances of ARG-carrying bacteria in the gut. While many different gut microbes encode for ARGs, they are not uniformly distributed across, or transmitted by, various members of the microbiome, and not all are of equal clinical relevance. Both experimental and theoretical approaches in microbial ecology have been applied to understand differing frequencies of ARG horizontal transfer between commensal microbes as well as between commensals and pathogens. In this commentary, we assess the evidence for the role of commensal gut microbes in encoding antimicrobial resistance genes, the degree to which they are shared both with other commensals and with pathogens, and the host and environmental factors that can impact resistome dynamics. We further discuss novel sequencing-based approaches for identifying ARGs and predicting future transfer events of clinically relevant ARGs from commensals to pathogens.

## Introduction: the constituents of the resistome

The combined abundances of all antibiotic resistance genes (ARGs) circulating in the gut, termed the gut “resistome”, is crucial to understand for two primary reasons. From a direct perspective, ARGs can determine how an individual’s gut microbiome will respond to and recover from antibiotic therapy, and are therefore important for predicting microbiome dynamics^1–3^ and antibiotic-associated bloom of pathobionts such as vancomycin-resistant *Enterococcus*.^1,2,4^ However, much attention is given to the resistome due to a proposed indirect effect: the possibility that commensal species may share their antimicrobial resistance genes with pathogens or pathobionts and therefore confer antimicrobial resistance to virulent and clinically relevant strains.^5^ Understanding the degree to which commensal microbes may contribute to antimicrobial resistance spread in pathogens is of pressing concern considering the global burden of antimicrobial resistance surpassed one million attributable deaths in 2019, and is projected to result in as many as ten million annual deaths by 2050.^6^ The frequency of these ARG transfer events likely depends on a multitude of factors, including the taxonomic and ecological similarity between donors and recipients, the classes of associated mobile elements, the distribution and abundances of ARGs within commensal microbes, and environmental conditions and stressors in the microbial niche. Thus, if antimicrobial usage selects for ARGs in commensal strains, and in turn increases their abundance, it may also increase the likelihood/frequency of ARG transfer to pathogenic strains.^7^ While unknowns remain surrounding the frequency and ecological relevance of this proposed interaction, the evidence provided below clearly show that many members of the human microbiome possess *bona fide* ARGs and commonly transfer them via horizontal gene transfer (HGT).

Some of the earliest evidence that the gut microbiome acts as a reservoir of ARGs was an observed increase between 1970 and 1997 in frequency of *tetQ* and *ermF* genes carried on conjugative transposons and plasmids from identified *Bacteroides* isolates.^5,8,9^ The possibility that commensals might be a reservoir of ARGs was further bolstered by the identification of *vanB* genes in vancomycin-resistant gut isolates of *Eggerthella lenta* and *Clostridium innocuum*.^10^ Large functional metagenomic screens, in which random gut microbial DNA libraries are screened for antimicrobial activity in a heterologous *Escherichia coli* host, enabled cultivation-independent high-throughput identification of ARGs within the human microbiome.^11^ ARGs have been readily identified across functional metagenomic screens from the *Bacteroides*,^11^ *Bifidobacterium*,^12^ *Clostridium*, and *Enterococcus*^13^ genera, as well as from several commensal Firmicutes.^14^ While Enterobacterales typically represent only a minority of the adult gut microbiome, functional screens have identified a large number of ARGs in this order in both aerobic cultivation libraries^11^ and metagenomic libraries from the infant gut,^13,15^ in which this bacterial order can be more abundant and encode a majority of ARGs.^16^ Sequencing and assembly of resistant clones can identify complete gene sequences that confer ARG resistance for hundreds of resistant clones simultaneously for high-throughput functional identification of ARGs.^17^ Functional metagenomics directly screens genes for resistance phenotypes and therefore this approach can identify novel ARGs with high specificity, and can be used for the development and augmentation of custom ARG databases.^18,19^ Through sequence homology to known ARGs, the widespread use of shotgun metagenomics has further enabled detailed analysis of mammalian-associated resistomes across body sites,^20–22^ demonstrating that the resistome displays inter-individual variation and correlates with a number of factors further discussed below, including diet,^23,24^ travel,^25^ antibiotic usage,^2,22,26,27^ hospital environments,^28^ and breastfeeding.^15,27,29^ Yet, determining the clinical relevance of a given ARG presence within the gut microbiome depends not just on its abundance, but also on the relative risks posed by different ARG classes and the frequencies by which they may transmit to pathogens or pathobionts.

## Who shares ARGs with whom?

The most scrutinized clinical attribute of the resistome in public health is its capacity to act as a reservoir of ARGs that may be acquired by pathogenic strains through HGT. In this ecological model, population-level changes in ARG abundances in commensal microbes might increase the frequency of successful transmission of these genes to pathogenic strains. However, while HGT is prolific among prokaryotes, it is clear that bacteria do not share genes equally. One analysis of HGT events identifiable across microbial genomes found that HGT is highly predicted by both phylogenetic similarity and ecological overlap.^30^ Closely related microbes are more likely to engage in gene transfer, as are microbes that coexist within the same niches – including the general human-associated niche and individual body sites.^30^ This has also been shown in the case of allelic exchange via homologous recombination, which can occur rampantly within some bacterial species; however, this occurs in an unequal and structured pattern that may represent shared ecologies within species.^31,32^ Because bacterial HGT is far from panmictic, species-specific models of transmission frequencies that build on both experimental and genomic evidence are tantamount for understanding the extent and risks of ARG HGT.

Both experimental and clinical evidence have firmly established that ARGs can be horizontally transferred between microbes *in vivo*. In experimental settings, spreading of ARGs via HGT between members of the Enterobacterales appears particularly frequent. While Enterobacterales are generally a minority in the healthy gut, in an inflamed state they can bloom in abundance and engage in highly frequent HGT.^7^ HGT of ARG plasmids between *Klebsiella pneumoniae* and *E. coli* has been observed in clinical cases.^33,34^ Another clinical example identified three Enterobacterales (*E. coli, K. pneumoniae*, and *Enterobacter cloacae*) co-infecting a patient that all possessed a blaOXA-48-harboring IncL/M-type plasmid, hinting that the plasmid had been acquired by two of the strains while within the patient’s gut microbiome.^35^ Conjugation of an ARG plasmid between *E. coli* strains has also been observed in the infant gut^36^ and in a human challenge experiment.^37^ Even in the absence of any antibiotic selective pressure, *Serratia liquefaciens* was observed transferring a plasmid with extensive ARGs to *E. coli* within the mouse gut.^38^ Additionally, post-antibiotic persisters of *Salmonella enterica* were found to easily transfer resistance plasmids to *E. coli* in the murine gut.^39^

Beyond Enterobacterales, experimental and clinical evidence have established a propensity for HGT of ARGs in *Enterococcus*. This genus is of particular clinical interest due to the global rise in vancomycin-resistant *Enterococcus* (VRE) responsible for a significant number of nosocomial infections.^40^ The population structure of *E. faecium* is known to be shaped by recombination,^41^ and a large fraction of its genome is composed of mobile genetic elements,^42^ indicating that HGT plays a significant role in the lifestyle of this species. In the gnotobiotic mouse gut, a genomic transposon containing a *vanB2* gene encoding for vancomycin resistance was observed to transfer from *Clostridium symbiosum* to *Enterococcus faecalis* and *E. faecium*, mirroring cross-species acquisitions of *vanB* genes in the emergence of VRE.^43^ Similarly, in a human challenge experiment, volunteers ingested both a vancomycin-resistant *E. faecium* isolated from chickens and an *E. faecium* isolate from the human microbiome, and transfer of the *vanA* gene was observed in 3/6 patients during the experiment.^44^

In circumstances where ARG transmission cannot be identified by experimental challenge or longitudinal sampling, genomics can provide evidence of recent or ancestral HGT events. The most straightforward identification of HGT events are genomic regions, transposons, or plasmids that are far more similar in nucleotide identity than the core genome (e.g., the *16s* gene) between two bacterial hosts. This implies a more recent ancestry of a genomic locus than would be expected by vertical inheritance. This can be observed in a case of a *Tn1546* transposon encoding vancomycin resistance genes first identified in *Staphylococcus aureus* in 2003 that was near-identical to the transposon in *E. faecalis*.^45^ In turn, a 94% nucleotide identity of the *Enterococcus vanA* gene to a copy in soil *Paenibacillus* identifies an environmental origin of the gene within epidemic VRE as well.^46^ Since then, copies of *vanA* genes have been circulating in *Enterococcus* with rampant inter-strain sharing of different versions of the gene^46,47^ and further transfer with gut commensals such as *Eggerthella lenta* and *Clostridium innocuum*.^10^ Thus, while the majority of HGT events do tend to occur within species, VRE provides an example of how once an inter-family ARG HGT event occurs, the gene can then proliferate quickly among strains of the recipient species and among other species that share a similar ecology. More generally, there is significant genomic evidence for frequent HGT among commensal *Bacteroides*,^48^ with near-identical mobile elements including erythromycin and tetracycline ARGs occurring across species from this genus.^8^ In contrast, within Proteobacteria (mostly Enterobacterales), phylogenetic inference of the origins of ARGs identified 22 originator strains for common ARGs, 21 of which were at least sometimes pathogenic.^49^ In part, this may be due to the noted preference of Proteobacteria to share genes mostly within class and phylum, and the predominance of facultative and opportunistic pathogens in this group. In sum, while HGT is near-universal among major groups of gut microbes, the degree and frequency to which microbes act as donors or recipients can be highly species-specific.

## Environmental and host factors shape the gut resistome

**Figure 1.**
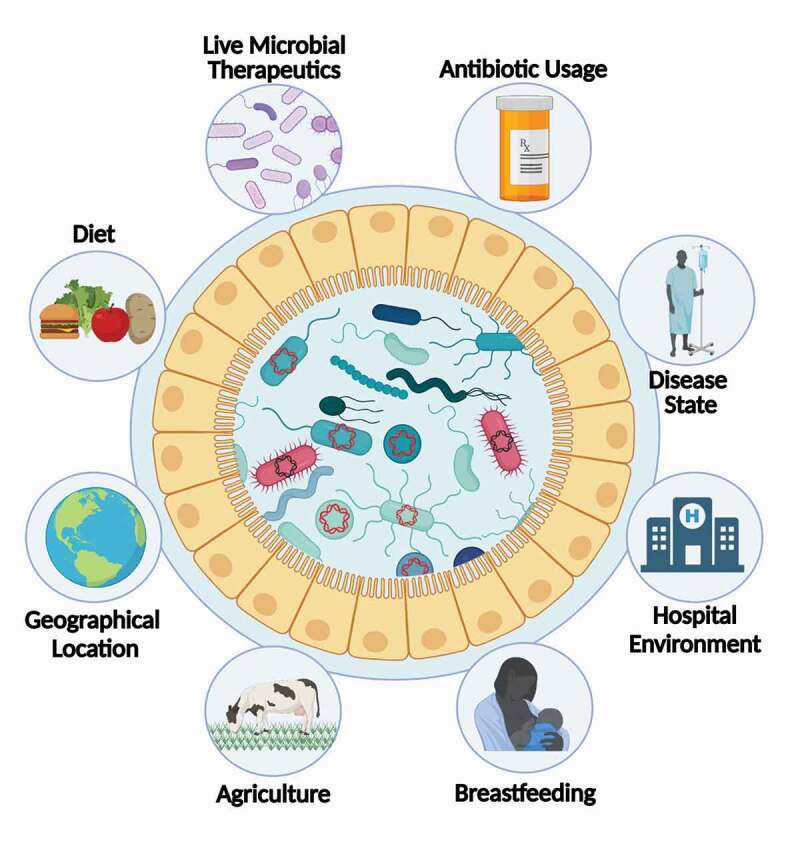
**Host and Environmental Factors that Impact the Gut Resistome**. The abundance and composition of the gut resistome are influenced by a multitude of factors. **(1) Live Microbial Therapeutics**; Live Microbial Therapeutics can reduce or expand the resistome depending on the environmental context, such as the administration of antibiotics. **(2) Antibiotics**; Antibiotic usage can select for resident antibiotic-resistant microbes and leads to an overall expansion of the resistome. **(3) Disease State**; Certain disease states, such as IBD, are correlated with the overall expansion of the resistome through boosting horizontal gene transfer. **(4) Hospital Environment**; Hospital settings provide an ideal environment for the dissemination of antibiotic resistance genes between patients. **(5) Breastfeeding**; Antibiotic resistance genes can be vertically transmitted through breastmilk. Breast milk is associated with a suppressive effect on the resistome of infants. **(6) Agriculture**; Working or living in close proximity to livestock serves as an exposure route of antimicrobial resistance genes. Additionally, antibiotic resistance genes can be acquired from food itself. **(7) Geographical Location**; Geographical location is correlated with variations in the resistome. Additionally, depending on the destination, international travel leads to changes in the composition of the resistome. **(8) Diet**; Antibiotic-resistance genes can be derived from food itself, which can be driven by use of antibiotics in livestock. Additionally, certain diets, such as ones high in whole grains, have been correlated with a reduction in the resistome.

Much like the gut microbiome as a whole, the abundance and composition of the gut resistome can be influenced by a multitude of factors (Figure 1). Although ARGs are mobile, their distribution is still largely constrained by phylogeny,^50^ and thus many factors that reshape the microbiome have the potential to have some corresponding effect on ARG abundances. While any changes to the resistome can potentially have clinical relevance, we can further categorize factors that influence ARG abundances as both direct, which involve selective pressure on the ARGs themselves, and indirect, which involve selective pressures unrelated to ARG genes that nonetheless change microbial composition in a manner that influences ARG abundances.

The human gut resistome is directly affected by the clinical use of antibiotics. As can be expected after the application of a strong antimicrobial selective pressure, the number of ARG types and their overall abundances in the microbiome are often elevated in response to antibiotic therapy,^2,15,22,26^ although sometimes this response is subtle and either antibiotic-specific^51^ or species-specific.^3^ This enrichment is observed in the context of a general significant drop in community diversity following antibiotic exposure, indicating a selective bottleneck of antibiotic-resistant or tolerant communities.^3,26^ Direct factors also impact the resistome at population scales, as changes in antibiotic usage in both humans and animal agriculture have been shown to reflect the distribution of ARGs across countries,^52^ a signal that is also likely reflected in individual acquisition of location-specific ARGs upon travel to different global regions.^25^ On a more local level, direct exposure to farms using antimicrobials has also been shown to reshape individuals’ gut resistomes.^53^ The phylogenetic rooting of major epidemic clones of VRE within lineages derived from domesticated animals coincides with the historical start of antibiotic usage in animal farming, implicating farm antibiotic usage in the rise of clinically important antibiotic-resistant gut bacteria.^54^ In addition, multidrug-resistant Proteobacteria have been isolated from farmland soils that encode for ARGs nearly genetically identical to some found in clinical isolates.^55^ Thus, agricultural use of antimicrobials can affect the resistome through direct exposure, as well as through introduction of antibiotics and antibiotic-resistant species through the food chain. The hospital is another local environment that likely impacts ARG abundances via direct selection, as high selective pressures for antimicrobial resistance in this environment can result in ARG transfer between strains causing nosocomial infections within a hospital over time.^28^ With this context, it is unsurprising that the prevalences of ARGs in commensal *Bacteroides* has increased in the guts of healthy people over the last several decades.^5^ Thus, selective pressure from antibiotics can clearly elevate the abundances of ARGs in the microbiome at individual, local, and global levels.

Indirect factors also substantially shape the resistome. Most of these factors consist of the host’s specific environmental stimuli, however recent work points to host genetics accounting for 25% of the variation in the resistome of a human cohort.^56^ Acquisition of ARGs within the human microbiome starts immediately after birth, as infants will be colonized by at least some bacteria encoding ARGs even in the absence of individual antibiotic exposure,^2^ and this assemblage is influenced by the vertical transmission of ARGs and mobile genetic elements through maternal breast milk.^15,27,29^ Interestingly, cessation of breastfeeding is correlated with the expansion of ARGs in the infant microbiome associated with the expansion of Enterobacterales.^29^ In older children, a high fiber dietary intervention significantly decreased the total abundance of ARGs, particularly those also found within the Enterobacterales.^24^ Other dietary elements such as artificial sweeteners have also been shown (in culture) to induce upregulation of conjugation machinery in response to cell stress that may result in increased ARG transfer.^23^ Diseases connected to gut inflammation have also been observed to lead to an expansion of the intestinal resistome, including type 2 diabetes, cirrhosis, obesity, and IBD.^7,24,57^ A common mechanism of increased ARG prevalence across these diseases is likely the blooming of ARG-enriched taxa such as the Enterobacterales in response to a dysbiotic state.^58^ Dietary and therapeutic interventions that reduce the abundance of Enterobacterales could therefore beneficially modulate the resistome.^24^ ARG expansions in pathobionts, including many members of the Enterobacterales, or in commensals with higher propensity to spread ARGs to pathobionts, may be considered higher risk events, and thus it is valuable to further identify the taxonomic basis for a given shift in ARG abundances. Of note, ARG carriage by commensals could be beneficial in some scenarios. Following a course of antibiotics, the reduction in microbiome diversity and functions facilitates colonization with bacterial pathogens. Commensals that are resistant to the administered antibiotic can provide colonization resistance against the invading pathogen and thus protect the host from disease.^59^ The extent to which this phenomenon can be mediated by ARG acquisition (rather than intrinsic resistance) remains to be determined.

## Expansion and diminution of the gut resistome by live microbial therapeutics

**Figure 2.**
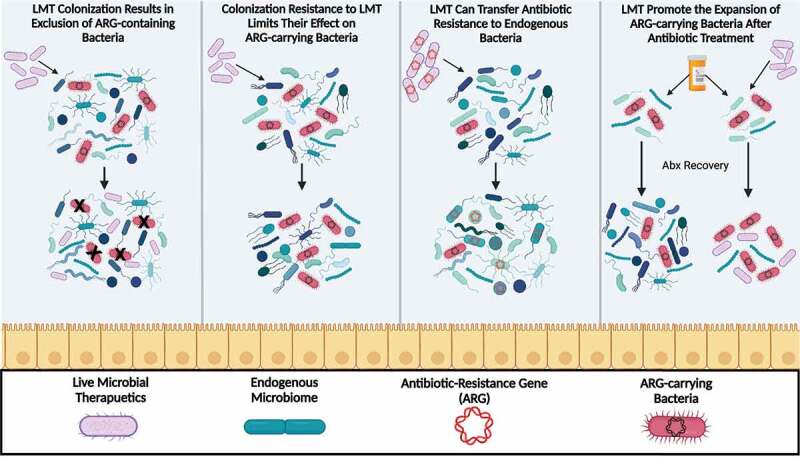
**Effects of Live Microbial Therapeutics (LMT) on the Gut Resistome**. When LMTs are administered, they can have a variety of consequences on gut commensals. Left to right; LMTs can directly reduce the overall number of ARG-carrying bacteria. However, this may depend on whether the LMTs are able to colonize the gut of the host. Colonization resistance to LMT may limit their effect on ARG-carrying bacteria. Second, LMTs containing ARGs can transfer antibiotic resistance to endogenous microbes. For example, many LMTs utilize lactic acid bacteria, which are known to be antibiotic-resistant, and are able to undergo horizontal gene transfer with commensal species. Finally, LMTs may indirectly promote expansion of the resistome, by supporting the bloom of ARG-carrying bacteria in the antibiotic-perturbed gut.

With both widespread commercial and clinical administration^60–62^ of live microbial therapeutics (LMT), including probiotic dietary supplements, the clinical usage of fecal microbiome transplantation (FMT), and the development of next-generation therapeutic microbial consortia, there is a pressing need to understand how these microbial supplements can affect the gut resistome.^63,64^ Probiotics and other LMT could affect the abundances of ARGs in the microbiome through either inhibitory or stimulatory effects on the abundances of other microbes in the gut (Figure 2). It has been suggested that probiotic supplementation may decrease the total load of ARGs within the gut.^65,66^ The current evidence for this effect is weak, with no probiotics-associated reduction in ARG abundances observed in some studies directly profiling the resistome^67^ or reanalyzed^22^ to determine the effect of probiotics on the resistome.^68,69^ Notably, a beneficial diminutive impact of probiotics on the resistome may depend on the ability of the exogenous bacteria to colonize the human gastrointestinal mucosa^22^ (Figure 2).

Several studies have observed a comparatively more substantial effect of FMT on reducing ARG load, including in patients with *C. difficile* infection,^70–73^ cirrhosis,^71,74^ and general colonization of antibiotic-resistance pathogens or pathobionts.^75^ The effectiveness of FMT in reducing ARG load in the gut across studies may be due primarily to a greater likelihood of colonization compared to most common probiotics. This in turn leads to a greater reduction in colonization of pathogens, pathobionts, and inflammation-associated microbial taxa present in dysbiotic states that can often carry a significant number of ARGs.^73^ In a direct comparison, FMT has been shown to alleviate ARG load while probiotic supplementation with Lactic Acid Bacteria (LAB) and *Bifidobacterium* increased ARG load by promoting the expansion of endogenous ARG-encoding microbes.^22^ While LMTs are not currently used for the sole purpose of resistome diminution, the impact of FMT in reducing ARG load in individuals in a dysbiotic state induced either by antibiotics or disease so far appears more reproducible, compared to common probiotics for which results can be mixed.

In addition to indirectly contributing to ARG expansion by microbiome modulation, LMT may impact the resistome directly by horizontally transferring the ARGs carried by the exogenous administered strains themselves (Figure 2). Many probiotics are LAB, and some of which can often be resistant to antibiotics,^76^ and may represent a risk for ARG transmission if not carefully monitored. There are a variety of studies that focus on determining the safety of these widely used LAB probiotics, specifically *Lactobacillus*,^77–79^ *Bacillus*,^64^ and nonpathogenic *Enterococcus*,^80^ with the purpose of identifying putative ARG or virulence-associated genes in commercial strains. Conjugative ARGs have indeed been identified in food-associated LAB. A *Lactococcus* species isolated from raw milk cheese was found to possess a multidrug-resistant plasmid that could be taken up by *E. faecalis in vitro*.^81^ In experimental settings, HGT between *Lactobacillus* and potential pathogens has been observed; wild-type strains of *Lactobacillus plantarum* isolated from fermented sausage were found to transfer plasmids containing the *tetM* and *ermB* resistance genes to *E. faecalis* in gnotobiotic mice.^82^ Furthermore, 50% of *Lactobacillus* strains isolated from sausage with *tetM* plasmids could transfer them to *E. faecalis in vitro*, and a smaller proportion could transfer them to the common probiotic *Lactococcus lactis*.^83^ Recent metagenomic analysis of both yogurt and kefir cultures have further identified a number of ARGs associated with mobile genetic elements in yogurt and kefir cultures.^84^ However, not all ARGs represent an equal clinical risk or risk of transmission. For example, a recent safety review of *Bacillus coagulans* GBI-30, strain 6086 identified several putative ARGs in its genome, but none of which were adjacent to or a part of mobile elements. Frameworks to prioritize ARGs into different risk groups may be helpful in evaluating the clinical ARG-related risks of genes found within a given strain,^85^ yet clinically novel or highly divergent ARGs may also represent an unknown risk that is difficult to quantify.^86^ While further genomic and experimental work is needed to understand the potential for probiotic strains to facilitate ARG transmission, there is still little evidence connecting it to direct transmission of clinically relevant ARGs. From a risk management perspective, the potential for ARG carriage and transfer by LMTs should be weighed against their clinical benefits in a given context.

## Metagenomic methods and challenges in identifying ARGs in the microbiome

While functional screens provide direct evidence for a gene’s involvement in antibiotic resistance, sequence homology is a powerful and commonly employed tool to identify ARGs within the human microbiome. This method only requires metagenomic sequencing, and can therefore be employed in a high throughput fashion on any metagenomic dataset.^13^ This has led to the development of an ecosystem of gene databases and software tools for the identification of ARGs within metagenomes.^87–92^ Linking ARGs and their associated mobile elements to their bacterial hosts in complex microbial communities is therefore the first step to understanding ARG transmission. One way to achieve this is with large-scale longitudinal sampling and sequencing of bacterial isolates from individuals,^93^ but the challenge of isolating and sequencing thousands of diverse microbial species is highly time-consuming and computationally intensive. With metagenomic assembly and binning, ARGs can be directly linked to their hosts either via assembly of the genomic region of interest, or by binning plasmids with host chromosomes. 58% of ARGs identified in wastewater sludge were found on plasmids rather than chromosomes, and metagenome-assembled genomes of *Burkholderiaceae* identified putative multidrug-resistant strains.^94^ However, metagenomic assemblies often represent only the more abundant fraction of a community, and mobile elements are considered the most difficult genomic regions to bin accurately in metagenomic assemblies, and thus for more accurate assignment of ARGs to hosts, additional methods are needed.^95^

Assigning *bona fide* function within the diverse, and often subtle, sequence-function space of ARGs and their homologs can prove difficult. The challenges with *in silico* prediction of ARG function have been illustrated by a cross-laboratory study in which authors found that participating labs often assigned discordant predictions of ARG even in isolates from well-characterized species.^96^ This was further demonstrated by a careful analysis of ARGs in metagenomic viral genomes, which found that homology thresholds sufficient for the identification of novel ARGs were simultaneously too loose to avoid a substantial number of false positives.^97^ The degree to which this is true is highly dependent on the class of ARG. For example, multidrug transporters that confer antibiotic resistance are generally subclasses of highly diverse superfamilies of proteins, such as ATP-binding cassette transporters or the major facilitator superfamily, that facilitate transport of an enormous range of substrates. Generalized prediction of the substrate specificity of these proteins is currently largely an unsolved problem,^98,99^ and the relationship between how sequence correlates with substrate(s) for these proteins is also only starting to be understood.^100^ Similarly, the diverse number of beta-lactamase homologs, with sometimes diverging functions, requires nuanced phylogenetic classification of sequences bounded by experimental characterization.^97^ This may also explain why predicted beta-lactamase homologs have failed to confer resistance when experimentally tested.^101^ Despite these limitations, strong correlations with phenotypic and clinical signals support the utility of sequence homology-based analyses of ARG for the interrogation of the abundance and distribution of ARGs in bacterial communities.^1–3,27,52,102^

Another limitation in gut resistome studies is the nearly-exclusive profiling of stool samples as a proxy for the intestinal community. However, stool samples can differ significantly in microbial composition from the gut itself, and these community differences can lead to differences in ARG detection and quantification. Commensals such as *Akkermansia muciniphila* and *Bacteroides* species are enriched within the mucosal layer, while *Bifidobacterium* species and *Eubacterium* species are most abundant within fecal samples,^22^ and these differences change the abundance and types of ARGs encountered in endoscopic samples compared to fecal samples.^22^ Most ARG classes were found to be under-enriched in stool compared to primary gut samples, and changes in ARG abundances observed in the GI tract were respectively not seen in stool samples. This was particularly true within the *Escherichia* genus enriched in the upper GI tract compared to stool, primarily due to species-specific location colonization preferences.^22^ Considering LAB probiotic species tend to colonize in these regions^103^ these findings also suggest that probiotic mediation of ARG composition may not be fully represented in stool samples. Due to these disparities, future studies tracking ARG abundance or transmission may necessitate inclusion of primary gut samples in addition to feces in order to fully capture ARG dynamics within the gut.

## Next-generation approaches to detect and predict ARG transmissions

A key goal for the antibiotic resistance field would be to identify ARG HGT events *in vivo* in a cultivation-independent and high-throughput manner. Hi-C proximity ligation in metagenomic samples is a new approach used to barcode sequencing reads by their physical proximity within cells, enabling computational association of mobile elements and plasmids with host genomes.^104,105^ Hi-C has recently been used to specifically interrogate the sharing of ARGs in the guts of healthy and immunodeficient patients, identifying putative HGT transmission events in the guts of all studied subjects on a timescale of less than two weeks.^106^ Specifically, many ARG transmission events were observed both within the Enterobacterales order, as well as inter-phylum HGT of ARGs, including the transfer of a plasmid encoding an efflux pump from the commensal *Blautia hansenii* to *Klebsiella*, and a plasmid with a multidrug efflux pump from *E. coli* to the commensal *Bacteroides* sp. A1C1. Deeply sequenced Hi-C samples combined with genome-resolved metagenomics will likely continue to be a powerful tool for the high-throughput detection of ARG transmission in complex communities.

In addition to identifying whether an ARG was horizontally acquired in retrospect, the ability to predict future ARG transmissions is a key goal in translating resistome research. Global surveillance of antimicrobial resistance in potentially high-risk environments, such as hospitals,^107^ animal sectors,^108^ wastewater,^109^ and rural communities^18^ can play a critical role in identifying ARGs and pathogens with recently acquired ARGs that may be poised for future epidemic spread. Genomics, isolate screens, and functional metagenomics can all be used for ARG surveillance. Other current predictive approaches are based on the assumption that rates of HGT between microbial groups will reflect future risk of ARG HGT.^110^ Furthermore, quantitative modeling shows that proximity to mobile genetic elements directly predicts the frequency of an ARG’s transfer. Thus, by identifying ARGs colocalized with mobile genetic elements in microbes with high frequencies of HGT with common pathogens, a list of ARGs at high risk for future emergence in pathogens can be developed.^110^ One such example, the beta lactamase gene *ctx-m-125*, is associated with four different mobile elements with broad host ranges and may therefore be poised for emergence in pathogens in which it has not currently been identified in.^110^ Furthermore, machine learning models can successfully predict rates of HGT between species and within strains of the same species based on the functional and metabolomic traits of the two genomes.^111^ Future directions for the field likely involve the incorporation of similar models into metagenomic resistome profiling, in which the resistome can be assessed and compared in terms of quantitative gene profiles and its predicted propensity for high-risk ARG transmission. Emergence of antimicrobial resistance in patients can also be predicted based on their past treatment history, clinical risk factors, and past colonization by resistant isolates.^112^ Future work may rely further upon predictions of patient risks for resistant infections using metagenomics of the human microbiome. Taken together, the use of new methodology can aid in developing more nuanced models of the resistome, moving the field from descriptors of the presence of ARGs toward an improved understanding of the risks and clinical relevance of ARG abundance and transmission in the human microbiome.
